# Intracranial and Extracranial Progression and Their Correlation With Overall Survival After Stereotactic Radiosurgery in a Multi-institutional Cohort With Brain Metastases

**DOI:** 10.1001/jamanetworkopen.2023.10117

**Published:** 2023-04-26

**Authors:** David J. Carpenter, Jim Leng, Muzamil Arshad, Will Giles, John P. Kirkpatrick, Scott R. Floyd, Steven J. Chmura, Joseph K. Salama, Julian C. Hong

**Affiliations:** 1 Department of Radiation Oncology, Duke University Medical Center, Durham, North Carolina; 2 Department of Radiation Oncology, University of Chicago Medical Center, Chicago, Illinois; 3 Department of Neurosurgery, Duke University Medical Center, Durham, North Carolina; 4 Radiation Oncology Clinical Service, Durham Veterans Affairs Health Care System, Durham, North Carolina; 5 Department of Radiation Oncology, University of California, San Francisco; 6 Bakar Computational Health Sciences Institute, University of California, San Francisco; 7 Joint Program in Computational Precision Health, University of California, San Francisco, and University of California, Berkeley

## Abstract

**Importance:**

Clinical trials for metastatic malignant neoplasms are increasingly being extended to patients with brain metastases. Despite the preeminence of progression-free survival (PFS) as a primary oncologic end point, the correlation of intracranial progression (ICP) and extracranial progression (ECP) events with overall survival (OS) is poorly understood for patients with brain metastases following stereotactic radiosurgery (SRS).

**Objective:**

To determine the correlation of ICP and ECP with OS among patients with brain metastases completing an initial SRS course.

**Design, Setting, and Participants:**

This multi-institutional retrospective cohort study was conducted from January 1, 2015, to December 31, 2020. We included patients who completed an initial course of SRS for brain metastases during the study period, including receipt of single and/or multifraction SRS, prior whole-brain radiotherapy, and brain metastasis resection. Data analysis was performed on November 15, 2022.

**Exposures:**

Non-OS end points included intracranial PFS, extracranial PFS, PFS, time to ICP, time to ECP, and any time to progression. Progression events were radiologically defined, incorporating multidisciplinary clinical consensus.

**Main Outcomes and Measures:**

The primary outcome was correlation of surrogate end points to OS. Clinical end points were estimated from time of SRS completion via the Kaplan-Meier method, while end-point correlation to OS was measured using normal scores rank correlation with the iterative multiple imputation approach.

**Results:**

This study included 1383 patients, with a mean age of 63.1 years (range, 20.9-92.8 years) and a median follow-up of 8.72 months (IQR, 3.25-19.68 months). The majority of participants were White (1032 [75%]), and more than half (758 [55%]) were women. Common primary tumor sites included the lung (757 [55%]), breast (203 [15%]), and skin (melanoma; 100 [7%]). Intracranial progression was observed in 698 patients (50%), preceding 492 of 1000 observed deaths (49%). Extracranial progression was observed in 800 patients (58%), preceding 627 of 1000 observed deaths (63%). Irrespective of deaths, 482 patients (35%) experienced both ICP and ECP, 534 (39%) experienced ICP (216 [16%]) or ECP (318 [23%]), and 367 (27%) experienced neither. The median OS was 9.93 months (95% CI, 9.08-11.05 months). Intracranial PFS had the highest correlation with OS (ρ = 0.84 [95% CI, 0.82-0.85]; median, 4.39 months [95% CI, 4.02-4.92 months]). Time to ICP had the lowest correlation with OS (ρ = 0.42 [95% CI, 0.34-0.50]) and the longest median time to event (median, 8.76 months [95% CI, 7.70-9.48 months]). Across specific primary tumor types, correlations of intracranial PFS and extracranial PFS with OS were consistently high despite corresponding differences in median outcome durations.

**Conclusions and Relevance:**

The results of this cohort study of patients with brain metastases completing SRS suggest that intracranial PFS, extracranial PFS, and PFS had the highest correlations with OS and time to ICP had the lowest correlation with OS. These data may inform future patient inclusion and end-point selection for clinical trials.

## Key Points

**Question:**

For patients completing stereotactic radiosurgery (SRS) for brain metastases, are intracranial and extracranial progression reliable surrogates for overall survival (OS)?

**Findings:**

In this cohort study of 1383 patients undergoing an initial course of SRS, both intracranial and extracranial progression-free survival were correlated with OS, with median durations approximately half that of OS.

**Meaning:**

These findings may support inclusion of patients with brain metastases completing SRS in oncologic clinical trials with composite primary outcomes.

## Teaser

This cohort study evaluates the correlation between intracranial and extracranial progression and overall survival after stereotactic radiosurgery among patients with brain metastases.

## Introduction

Nearly 30% of patients with solid tumors develop brain metastases.^1,2^ Up-front surgical resection and/or stereotactic radiotherapy (SRS) is an established standard of care for a majority of patients with a limited number of brain metastases.^3,4^ Historically, patients with brain metastases were excluded from clinical trials of systemic therapies for metastatic malignant neoplasms owing to poor overall prognosis. However, due to marked improvements in overall survival (OS) among patients with brain metastases^5,6,7,8^ and improved intracranial response rates among systemic agents,^9,10,11^ an increasing proportion of patients with brain metastases are being enrolled in randomized trials of systemic agents.^2^

Oncologic trials have failed to demonstrate therapeutic benefits more often than trials for nononcologic conditions.^12^ This discrepancy is often attributed to challenges in estimating oncologic treatment benefit through accurate design assumptions.^13^ Inclusion of patients with brain metastases may introduce several additional challenges to trial design. Eligibility criteria commonly utilize standardized radiologic assessment and OS prognostication.^5,6,14^ However, response assessment may differ between patients with newly diagnosed brain metastases and those receiving up-front intracranial metastasis-directed therapy. Furthermore, progression-free survival (PFS) recently surpassed OS as the most common primary end point across randomized oncologic trials.^15^ While OS prognostication is well established for patients with brain metastases,^5,6,7^ risk of intracranial progression (ICP) is less defined.^16,17,18^

The NRG Oncology BN009 trial demonstrates the utility of incorporating the association between ICP and OS into clinical trial design.^19,20^ However, aside from brain metastasis velocity,^19^ the correlations of ICP and extracranial progression (ECP) with OS remain poorly understood. Better understanding of these correlations may inform several aspects of clinical trial design, including inclusion criteria, composite end-point selection, and assessment time frames.^21^ To this end, we analyzed a large multi-institutional cohort of patients with brain metastases completing SRS to report the correlation of ICP and ECP end points with OS.

## Methods

This retrospective cohort study was approved by the Duke University Medical Center Institutional Review Board and an informed consent waiver was granted because deidentified data were used. The study followed the Strengthening the Reporting of Observational Studies in Epidemiology (STROBE) reporting guideline.

We identified all adult patients at Duke University Medical Center and the University of Chicago Medical Center who completed an initial course of SRS for brain metastases between January 1, 2015, and December 31, 2020. This time frame was selected to reflect contemporary multidisciplinary brain metastasis management practices, including receipt of immune checkpoint and molecularly targeted or small-molecule inhibitor therapies. Permissible cases included single and/or multifraction SRS, including those with prior whole-brain radiotherapy or brain metastasis resection. Demographic, clinical, and treatment-specific parameters were obtained via record review. Oligometastatic disease burden was defined as 1 to 5 metastatic (ie, non-locoregional) lesions present across all anatomic locations, including intracranial disease, at the time of SRS.^22^

Dates of initial cancer diagnosis and progressive disease were manually obtained from pathology records (where available), radiology images and reports,^14^ and multidisciplinary clinical consensus. All time-to-event outcomes were calculated from the time of SRS completion. We defined ICP as any clinical concern for local or distant brain metastasis progression; time to ICP (patients censored at death) and intracranial PFS (death recorded as an event) were estimated from time of SRS completion. Time to ECP and extracranial PFS were estimated using a similar approach. Time to any progression and PFS incorporated both ICP and ECP events.

Data on patient race and ethnicity were obtained via self-report to contextualize the patient population completing SRS as well as to assess correlation to clinical outcomes. Patients self-identified as Black, White, or other or unknown. Other or unknown refers to any patients who chose to self-identify as any race other than Black or White, including those who preferred not to answer (unknown).

### Statistical Analysis

Clinical end points were estimated using the Kaplan-Meier method. The correlation (ρ [95% CI]) between OS and other clinical end points was measured using normal scores rank correlation with the iterative multiple imputation approach for analysis of correlations between 2 partially censored failure times.^23^ This rank correlation method allows for analysis of associations between 2 times to event, such as survival times and surrogate end points of time to ICP or time to ECP, while accommodating for censoring of variates of interest. Using these methods, end-point correlation with OS was measured across all patients with brain metastases as well as for those with primary tumors of all non–small cell lung (NSCLC), driver-mutated NSCLC, breast, and melanoma (including noncutaneous) origin. Sensitivity analyses of end-point correlation were performed for 2 subpopulations: (1) for patients with both intracranial and extracranial imaging obtained following SRS and (2) for patients who experienced ICP and/or ECP. Finally, among the latter subpopulation, logistic regression was performed to identify clinical parameters associated with initial post-SRS progression at an intracranial vs extracranial location. Multivariate logistic regression was performed for all variables with *P* < .05 on univariate analysis. Analyses were performed using R, version 4.1.2 (R Project for Statistical Computing), including the SurvCorr R package, version 1.0 (R Project for Statistical Computing), for the computation of correlations between OS and other clinical end points of interest. Data analysis was performed on November 15, 2022.

## Results

In this cohort study, we identified 1383 patients across 2 institutions (Table 1). There were 758 women (55%) and 625 men (45%). At the time of SRS, the mean patient age was 63.1 years (range, 20.9-92.8 years). A total of 1002 patients (73%) had a Karnofsky performance status score of 80 or greater. In terms of race and ethnicity, 283 patients (20%) were Black, 1032 (75%) were White, or 68 (5%) were other or unknown race and ethnicity. Common primary tumor sites included the lung (757 [55%]), breast (203 [15%]), and skin (melanoma; 100 [7%]). Prior to SRS, 893 patients (65%) underwent systemic therapy, 361 (26%) underwent brain metastasis resection, and 142 (10%) underwent prior whole-brain radiotherapy. At the time of SRS, 995 patients (72%) had extracranial disease, 470 (34%) had controlled extracranial disease, and 648 (47%) had oligometastatic disease. Multiple brain metastases were present in 735 patients (53%); the mean (SD) planned target volume (PTV) was 12.5 (17.5) cc for the largest single brain metastasis and 15.4 (20.1) cc for all irradiated brain metastases within a single patient. Following SRS, 938 patients (68%) underwent any systemic therapy, including immune checkpoint (455 [33%]) and molecularly targeted or small-molecule inhibitor (361 [26%]) therapies.

**Table 1.** Patient Demographic, Clinical, and Treatment Characteristics^a^

Characteristic	Patients (N = 1383)
Institution study population	
1	162 (12)
2	1221 (88)
Age at SRS, y, mean (range) [median (range)]	63.1 (20.9-92.8) [64.3 (55.4-72.1)]
Karnofsky performance status score	
100	123 (9)
90	508 (37)
80	371 (27)
70	227 (16)
60	78 (6)
≤50	76 (5)
Sex	
Female	758 (55)
Male	625 (45)
Race and ethnicity	
Black	283 (20)
White	1032 (75)
Other or unknown^b^	68 (5)
Primary tumor site	
Breast	203 (15)
Lung	757 (55)
Skin (melanoma)	100 (7)
Gastrointestinal	102 (7)
Gynecologic	33 (2)
Genitourinary	109 (8)
Head and neck	28 (2)
Other or unknown	51 (4)
Extracranial disease present at time of SRS?	
Yes	995 (72)
No	388 (28)
Total No. of extracranial metastatic sites at SRS, median (IQR)	2 (0-2)
Any site involvement^c^	
Lung	623 (45)
Node	482 (35)
Bone	470 (34)
Liver	272 (20)
Adrenal	163 (12)
Other sites	170 (12)
Metastatic burden at time of SRS	
Oligometastatic	648 (47)
Polymetastatic	735 (53)
Extracranial disease control at time of SRS	
Yes	470 (34)
No or unknown	913 (66)
No. of lines of pre-SRS treatment	
Systemic therapy	
0	490 (35)
1	343 (25)
2-3	348 (25)
≥4	202 (15)
Chemotherapy	
0	690 (50)
1	406 (29)
2-3	223 (16)
≥4	64 (5)
Immunotherapy	
0	1056 (76)
1	242 (17)
2-3	78 (6)
≥4	7 (1)
Targeted or small-molecule inhibitor therapy	
0	1079 (78)
1	166 (12)
2-3	119 (9)
≥4	19 (1)
Time from brain metastasis diagnosis to local intracranial therapy, d	
Within 21	852 (62)
22-60	341 (25)
>60	190 (14)
Prior resection	
Yes	361 (26)
No	1022 (74)
Prior whole-brain radiotherapy	
Yes	142 (10)
No	1241 (90)
No. of metastases at time of SRS	
1	645 (47)
2	247 (18)
3-5	308 (22)
≥6	183 (13)
Total PTV of all lesions, mean (SD), cc	15.4 (20.1)
PTV of largest lesion, mean (SD), cc	12.5 (17.5)
No. of lines of post-SRS treatment	
Systemic therapy	
0	445 (32)
1	489 (35)
2 to 3	367 (27)
≥4	82 (6)
Chemotherapy	
0	873 (63)
1	318 (23)
2-3	176 (13)
≥4	16 (1)
Immunotherapy	
0	928 (67)
1	388 (28)
2-3	67 (5)
≥4	0 (0)
Targeted or small-molecule inhibitor therapy	
0	1022 (74)
1	257 (19)
2-3	87 (6)
≥4	17 (1)

Abbreviations: PTV, planned target volume; SRS, stereotactic radiosurgery.

^a^ Unless indicated otherwise, values are presented as No. (%) of patients.

^b^ Includes patients who chose to self-identify as any race other than Black or White (ie, other), including those who preferred not to answer (ie, unknown).

^c^ Involvement across sites is not mutually exclusive; therefore, totals will exceed patient numbers.

A total of 383 patients (28%) were alive at the last follow-up (eTable in Supplement 1), and the median follow-up was 8.72 months (IQR, 3.25-19.68 months). Intracranial progression was observed in 698 patients (50%), preceding 492 of 1000 observed deaths (49%) (eTable in Supplement 1). Extracranial progression was observed in 800 patients (58%), preceding 627 of 1000 observed deaths (63%) (eTable in Supplement 1). Irrespective of deaths, 482 patients (35%) experienced both ICP and ECP, 534 (39%) experienced ICP (216 [16%]) or ECP (318 [23%]), and 367 (27%) experienced neither (eTable in Supplement 1). Among the 258 patients (19%) who died in the absence of documented ICP or ECP, the median survival was 2.2 months (IQR, 1.97-4.15 months).

The correlation of OS with intracranial PFS, extracranial PFS, and PFS is presented in the Figure. The correlation of OS with time to ICP, time to ECP, and time to any progression is provided in the eFigure in Supplement 1. For all patients, median OS was 9.93 months (95% CI, 9.08-11.05 months) (Table 2). Across all non-OS end points, intracranial PFS had the highest correlation with OS (ρ = 0.84 [95% CI, 0.82-0.85]; median, 4.39 months [95% CI, 4.02-4.92 months]). Time to ICP had both the lowest correlation with OS (ρ = 0.42 [95% CI, 0.34-0.50]) and the longest median time to event (median, 8.76 months [95% CI, 7.70-9.48 months]). Progression-free survival had the shortest median time to event (ρ = 0.76 [95% CI, 0.73-0.78]; median, 3.07 months [95% CI, 2.97-3.20 months]).

**Figure.**
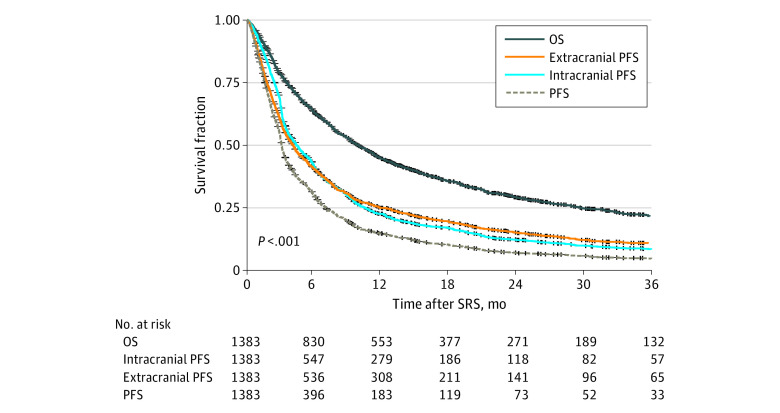
Correlation of Overall Survival (OS) With Intracranial Progression-Free Survival (PFS), Extracranial PFS, and PFS SRS indicates stereotactic radiosurgery.

**Table 2.** Correlations Between Clinical End Points and Overall Survival by Primary Tumor Type

End point	Median (95% CI), mo	ρ (95% CI)
All patients (N = 1383)		
Overall survival	9.93 (9.08-11.05)	[Reference]
Time to ICP	8.76 (7.70-9.48)	0.42 (0.34-0.50)
Intracranial PFS	4.39 (4.02-4.92)	0.84 (0.82-0.85)
Time to ECP	6.71 (6.12-7.70)	0.59 (0.55-0.63)
Extracranial PFS	4.12 (3.66-4.45)	0.81 (0.79-0.83)
Time to any progression	3.83 (3.46-4.32)	0.58 (0.52-0.64)
PFS	3.07 (2.97-3.20)	0.76 (0.73-0.78)
NSCLC (n = 674)		
Overall survival	10.96 (9.28-12.33)	[Reference]
Time to ICP	9.77 (8.77-12.14)	0.44 (0.32-0.55)
Intracranial PFS	5.08 (4.39-5.96)	0.85 (0.82-0.87)
Time to ECP	9.05 (7.11-10.93)	0.62 (0.53-0.69)
Extracranial PFS	4.86 (4.25-5.90)	0.83 (0.80-0.86)
Time to any progression	4.94 (4.22-5.87)	0.64 (0.58-0.70)
PFS	3.45 (3.20-3.99)	0.79 (0.76-0.82)
Driver-mutated NSCLC (n = 137)		
Overall survival	19.67 (13.48-32.96)	[Reference]
Time to ICP	9.51 (7.67-17.89)	0.43 (0.16-0.64)
Intracranial PFS	7.11 (5.76-8.88)	0.76 (0.66-0.84)
Time to ECP	9.25 (7.53-14.52)	0.63 (0.44-0.76)
Extracranial PFS	7.23 (6.00-9.26)	0.78 (0.69-0.85)
Time to any progression	5.97 (4.62-7.11)	0.59 (0.41-0.72)
PFS	4.86 (3.57-6.39)	0.71 (0.59-0.80)
Breast (n = 203)		
Overall survival	15.19 (10.93-19.98)	[Reference]
Time to ICP	6.85 (5.93-9.60)	0.47 (0.25-0.63)
Intracranial PFS	4.97 (4.35-5.93)	0.78 (0.70-0.83)
Time to ECP	5.44 (4.45-8.74)	0.64 (0.49-0.75)
Extracranial PFS	4.32 (3.60-5.46)	0.80 (0.72-0.85)
Time to any progression	3.61 (3.00-4.65)	0.63 (0.50-0.73)
PFS	3.11 (2.94-4.09)	0.72 (0.63-0.79)
Melanoma (n = 118)		
Overall survival	9.65 (7.83-13.06)	[Reference]
Time to ICP	5.86 (3.54-8.49)	0.47 (0.21-0.67)
Intracranial PFS	3.24 (2.98-4.48)	0.77 (0.68-0.84)
Time to ECP	5.27 (3.76-7.77)	0.61 (0.40-0.75)
Extracranial PFS	3.40 (3.03-5.33)	0.80 (0.71-0.86)
Time to any progression	3.07 (2.87-3.76)	0.57 (0.39-0.71)
PFS	2.87 (2.29-3.14)	0.73 (0.63-0.81)

Abbreviations: ECP, extracranial progression; ICP, intercranial progression; NSCLC, non–small cell lung cancer; PFS, progression-free survival.

Across subgroups by primary tumor type, median OS was greatest among the 137 patients with driver-mutated NSCLC (19.67 months [95% CI, 13.48-32.96 months]) and least among the 118 patients with melanoma (9.65 months [95% 7.83-13.06 months]) (Table 2). Within the NSCLC subgroup, correlation coefficients were similar to those across all patients: intracranial PFS had the highest correlation with OS, while time to ICP had the longest non-OS median time to event as well as the lowest correlation with OS. In contrast, among the driver-mutated NSCLC, melanoma, and breast subgroups, extracranial PFS had the highest correlation with OS (ρ = 0.78 [95% CI, 0.69-0.85]; median, 7.23 months [95% CI, 6.00-9.26 months]). In this study, PFS had a high correlation (ρ > 0.71 [95% CI, 0.59-0.80]) with OS across all primary tumor subgroups.

The first sensitivity analysis included 990 patients with both intracranial and extracranial surveillance imaging following SRS completion (393 of 1383 patients were excluded, including 287 with no post-SRS intracranial imaging and 359 with no post-SRS extracranial imaging) (Table 3). Among this subpopulation, the median OS was 16.88 months (95% CI, 15.16-19.00 months). Extracranial PFS had the highest correlation with OS (ρ = 0.72 [95% CI, 0.69-0.76]; median, 6.56 months [95% CI, 6.00-7.17 months]). Time to ICP had the lowest correlation with OS (ρ = 0.56 [95% CI, 0.49-0.61]; median, 8.03 months [95% CI, 6.91-8.95 months]).

**Table 3.** Correlations Between Clinical End Points and Overall Survival for Selected Subgroups of Interest

End point	Median (95% CI), mo	ρ (95% CI)
Both intracranial and extracranial imaging after SRS (n = 990)		
Overall survival	16.88 (15.16-19.00)	[Reference]
Time to ICP	8.03 (6.91-8.95)	0.56 (0.49-0.61)
Intracranial PFS	6.42 (6.03-7.06)	0.70 (0.66-0.73)
Time to ECP	7.17 (6.42-8.03)	0.63 (0.58-0.68)
Extracranial PFS	6.56 (6.00-7.17)	0.72 (0.69-0.76)
Time to any progression	4.15 (3.69-4.73)	0.62 (0.57-0.66)
PFS	4.12 (3.65-4.59)	0.65 (0.61-0.69)
ICP or ECP before death (n = 864)		
Overall survival	15.35 (13.86-17.28)	[Reference]
Time to ICP	6.23 (5.96-7.02)	0.52 (0.46-0.58)
Intracranial PFS	5.87 (5.40-6.19)	0.65 (0.60-0.69)
Time to ECP	5.74 (5.01-6.42)	0.62 (0.57-0.66)
Extracranial PFS	5.46 (4.86-6.20)	0.68 (0.64-0.72)
Time to any progression	3.41 (3.17-3.66)	0.60 (0.55-0.65)
PFS	NA	NA

Abbreviations: ECP, extracranial progression; ICP, intercranial progression; NA, not applicable; PFS, progression-free survival; SRS, stereotactic radiosurgery.

The second sensitivity analysis was limited to 864 patients with ICP and/or ECP following SRS (an additional 126 patients who did not have either documented ICP or ECP were excluded) (Table 3). Among this subpopulation, the median OS was 15.35 months (95% CI, 13.86-17.28 months). Extracranial PFS had the highest correlation with OS (ρ = 0.68 [95% CI, 0.64-0.72]; median, 5.46 months [95% CI, 4.86-6.20 months]), while time to ICP demonstrated the lowest correlation with OS (ρ = 0.52 [95% CI, 0.46-0.58]; median, 6.23 months [95% CI, 5.96-7.02 months]). Number of brain metastases (single vs 2 or vs 3-5 or vs ≥6) and volume of treated disease (either in terms of combined PTV of all treated brain metastases or PTV of the largest treated brain metastasis in a single patient) were not associated with initial patterns of progression. On multivariable logistic regression, ICP as an initial progression event was associated with oligometastatic disease at time of SRS (odds ratio [OR], 1.92 [95% CI, 1.42-2.59]; *P* < .001) (Table 4), while ECP as an initial progression event was associated with receipt of post-SRS chemotherapy (OR, 0.41 [95% CI, 0.30-0.55]; *P* < .001), immunotherapy (OR, 0.53 [95% CI, 0.39-0.72]; *P* < .001), and targeted therapy (OR, 0.58 [95% CI, 0.41-0.82]; *P* = .002).

**Table 4.** Univariate and Multivariate Logistic Regression^a^

Characteristic	Univariate analysis		Multivariate analysis^b^	
	OR (95% CI)	*P* value	OR (95% CI)	*P* value
Karnofsky performance status score ≥90 vs <90	1.26 (0.96-1.65)	.09	NA	NA
Age, per year	1.00 (0.98-1.01)	.42	NA	NA
Male sex	0.91 (0.70-1.20)	.52	NA	NA
White race	1.02 (0.75-1.39)	.90	NA	NA
Primary tumor type				
NSCLC driver vs NSCLC nondriver	0.93 (0.59-1.45)	.74	NA	NA
Breast vs NSCLC nondriver	1.00 (0.68-1.48)	>.99	NA	NA
Melanoma vs NSCLC nondriver	1.31 (0.80-2.14)	.28	NA	NA
Other vs NSCLC nondriver	0.71 (0.50-1.01)	.06	NA	NA
Controlled extracranial disease at time of SRS	1.54 (1.17-2.03)	.002^c^	1.07 (0.78-1.48)	.67
Oligometastatic disease at time of SRS	2.27 (1.73-2.99)	<.001^c^	1.92 (1.42-2.59)	<.001^c^
Receipt of pre-SRS treatment				
Chemotherapy	0.96 (0.74-1.26)	.78	NA	NA
Immunotherapy	1.05 (0.77-1.44)	.75	NA	NA
Targeted therapy	0.69 (0.50-0.95)	.022^c^	0.79 (0.55-1.13)	.20
Receipt of post-SRS treatment				
Chemotherapy	0.41 (0.31-0.54)	<.001	0.41 (0.30-0.55)	<.001^c^
Immunotherapy	0.63 (0.47-0.84)	.001^c^	0.53 (0.39-0.72)	<.001^c^
Targeted therapy	0.60 (0.44-0.81)	.001^c^	0.58 (0.41-0.82)	.002^c^
Brain metastasis resection	1.34 (1.00-1.81)	.06	NA	NA
Prior whole-brain radiotherapy	1.42 (0.90-2.25)	.14	NA	NA
Planned target volume, per cc				
All	1.01 (1.00-1.01)	.14	NA	NA
Maximum	1.01 (1.00-1.01)	.19	NA	NA
No. of brain metastases				
2 vs 1	0.91 (0.63-1.32)	.62	NA	NA
3-5 vs 1	1.27 (0.90-1.79)	.18	NA	NA
≥6 vs 3	1.22 (0.81-1.84)	.34	NA	NA
Time to initial progression event, mo	1.01 (0.99-1.03)	.17	NA	NA

Abbreviations: NA, not applicable; NSCLC, non–small cell lung cancer; OR, odds ratio; SRS, stereotactic radiosurgery.

^a^ For all 864 patients with either intracranial or extracranial progression, ORs are provided for parameters associated with intracranial (OR >1) vs extracranial progression (OR <1) as an initial site of post-SRS progression.

^b^ Only variables with *P* < .05 significance on the univariate analysis were included in the multivariate analysis.

^c^ *P* < .05.

## Discussion

In this large, multi-institutional, contemporary cohort study of patients with brain metastases completing an initial course of SRS, intracranial PFS, extracranial PFS, and PFS were associated with OS. Several key findings from this analysis may support judicious trial inclusion of patients with brain metastases while informing composite end-point selection and assessment time frames. The disproportionately high rate of failure among oncologic vs nononcologic trials reflects a lack of tools for specifying accurate design assumptions to realistically estimate treatment benefit.^12,13^ To this end, these data address 2 trends among oncologic trials: (1) increasing inclusion of patients with brain metastases in light of corresponding improvements in OS^9,10,11,24,25^ and (2) increasing use of composite clinical outcomes, such as PFS and intracranial PFS, as primary trial end points.^15^

For patients with brain metastases completing an initial SRS course, comparative rates of ECP, ICP, and death are poorly characterized. We observed that following an initial SRS course, patients were more likely to progress extracranially (58%) than intracranially (50%), with progression in a single location (ie, ICP or ECP) more common than progression at both sites (38% vs 35%). Patients most likely to have initial progression intracranially rather than extracranially were those with oligometastatic disease and those who did not receive any post-SRS systemic therapy (ie, chemotherapy, immunotherapy, or targeted therapy). As observed in a previous multi-institutional pooled analysis, disease burden and receipt of systemic therapy additionally demonstrate correlation with PFS among patients with exclusively extracranial metastases.^26^ Notably, we observed that initial patterns of progression were not associated with intracranial burden at time of SRS, with respect to either the number of brain metastases or the volume of treated disease. Finally, the higher likelihood of ECP among patients receiving post-SRS systemic therapy presumably reflects selection bias related to systemic therapy eligibility after patients are treated for their intracranial disease with SRS, rather than anticipated rates of intracranial vs extracranial control of specific systemic therapies.

Among patients who died in the absence of documented progression (19% of deaths), the relatively poor survival rate (median survival of 2.2 months) likely suggests a high proportion of undocumented progression (ie, patients experiencing rapid decline who died before being able to complete imaging that would presumably confirm progressive disease). A clinical trial population for which inclusion criteria mirror SRS eligibility might demonstrate a similar degree of ascertainment bias. Moreover, stricter inclusion criteria incorporating validated, site-specific prognostication tools^5,14,27,28^ would likely reduce the proportion of deaths in the absence of progression, thereby mitigating ascertainment bias.

Comparison across composite end points provides valuable context for trial end-point selection and assessment time frames.^21^ As a surrogate outcome for OS, PFS is now the most common end point used in clinical trials for patients with metastatic malignant neoplasms.^15^ Meanwhile, intracranial PFS is increasingly used as a primary end point among oncologic trials of systemic agents with favorable intracranial response rates.^29,30^ In this study, PFS had a high correlation with OS across all primary tumor subgroups. Both intracranial and extracranial PFS demonstrated higher OS correlation than PFS; however, whether intracranial or extracranial PFS had the highest correlation with OS appeared to differ across primary tumor origin.

Time-to-event outcomes, including time to ICP, have proved valuable among patients with brain metastases as a correlate to assess quality of life and neurologic decline, to determine optimal systemic therapy sequencing, and to tailor posttreatment surveillance.^16,31,32,33^ However, compared with event-free survival, time-to-event outcomes introduce substantial bias through censorship of mortality events.^34^ Accordingly, time-to-event outcomes are not as well defined as event-free and OS outcomes for patients with brain metastases completing an initial SRS course. Across all subpopulations in this study, time to ICP was numerically greater than time to ECP. However, neither proved to be a reliable OS surrogate, with a wide variance of median durations with respect to median OS. For example, time to ICP and time to ECP were nearly identical among patients with NSCLC and those with driver-mutated NSCLC, despite an almost 2-fold increase in median OS for driver-mutated cases. These data argue against the association between time-to-event outcomes and brain metastasis prognostication. This lack of correlation likely reflects ongoing advances in systemic therapy response rates. These data suggest that time-to-event outcomes should not be used as a surrogate outcome for OS among patients with brain metastases and should be reserved for analyses unrelated to OS in this population.

### Strengths and Limitations

Strengths of this cohort study include its large, multi-institutional population and corresponding event rates, which enabled robust analysis of correlation across a range of end points and primary tumor types. Potential limitations include generalizability outside of multidisciplinary brain metastasis–specific practices within large academic centers. Additionally, regardless of institution type, generalizability to specific clinical trial populations may be limited due to this study’s eligibility criteria for enrollment of patients at the time of initial SRS (vs time of initial brain metastasis diagnosis or time of potential trial enrollment). It is difficult to quantify potential follow-up bias resulting from the proportion of patients completing SRS followed by post-SRS management outside of identifiable electronic health record documentation. Because ICP and ECP documentation requires radiologic assessment, it is presumed that a proportion of patients died in the context of undocumented progression having not completed imaging. This is likely reflected in the relatively low median OS among deceased patients without progression, which was lower than PFS across all patients. Thus, mortality appears to be a primary driver of early composite outcomes, particularly within 3 months following SRS. Nevertheless, sensitivity analysis of those experiencing progression prior to death showed persistently high correlation of intracranial PFS, extracranial PFS, and PFS with OS. Finally, these data reflect patients completing an initial SRS course and therefore are not generalizable to those with progressive intracranial disease following SRS.

## Conclusions

The findings of this cohort study including patients with brain metastases completing an initial SRS course suggest that intracranial PFS, extracranial PFS, and PFS were highly correlated with OS across primary tumor types. For patients with brain metastases with post-SRS progression, initial progression at an intracranial rather than an extracranial location was associated with oligometastatic burden and lack of post-SRS systemic therapy. These data may inform clinical trial design for patients with brain metastases, including inclusion criteria and judicious selection of composite end points.

## Notes

Supplement 1. **eFigure.** Correlation of Overall Survival (OS) to Time to Intracranial Progression (TTICP), Time to Extracranial Progression (TTECP), and Time to Any Progression (TTP) **eTable.** Crude Event Rates for Death, Intracranial Progression (ICP), and Extracranial Progression (ECP) Across All Patients

Supplement 2.
Data Sharing Statement
