# Supplementary material for: Intracranial and Extracranial Progression and Their Correlation With Overall Survival After Stereotactic Radiosurgery in a Multi-institutional Cohort With Brain Metastases
Source: JAMA Netw Open. 2023 Apr 26;6(4):e2310117. doi: 10.1001/jamanetworkopen.2023.10117 (PMC10134007; doi:10.1001/jamanetworkopen.2023.10117)
Supplement: Supplement 1. — eFigure. Correlation of Overall Survival (OS) to Time to Intracranial Progression (TTICP), Time to Extracranial Progression (TTECP), and Time to Any Progression (TTP) eTable. Crude Event Rates for Death, Intracranial Progression (ICP), and Extracranial Progression (ECP) Across All Patients [file jamanetwopen-e2310117-s001.pdf]

Supplementary Online Content

Carpenter DJ, Leng J, Arshad M, et al. Intracranial and extracranial progression and their correlation with overall survival after stereotactic radiosurgery in a multi-institutional cohort with brain metastases. *JAMA Netw Open*. 2023;6(4):e2310117. doi:10.1001/jamanetworkopen.2023.10117

eFigure. Correlation of Overall Survival (OS) to Time to Intracranial Progression (TTICP), Time to Extracranial Progression (TTECP), and Time to Any Progression (TTP)

eTable. Crude Event Rates for Death, Intracranial Progression (ICP), and Extracranial Progression (ECP) Across All Patients

This supplementary material has been provided by the authors to give readers additional information about their work.

© 2023 Carpenter DJ et al. *JAMA Network Open*.

eFigure. Correlation of Overall Survival (OS) to Time to Intracranial Progression (TTICP), Time to Extracranial Progression (TTECP), and Time to Any Progression (TTP)

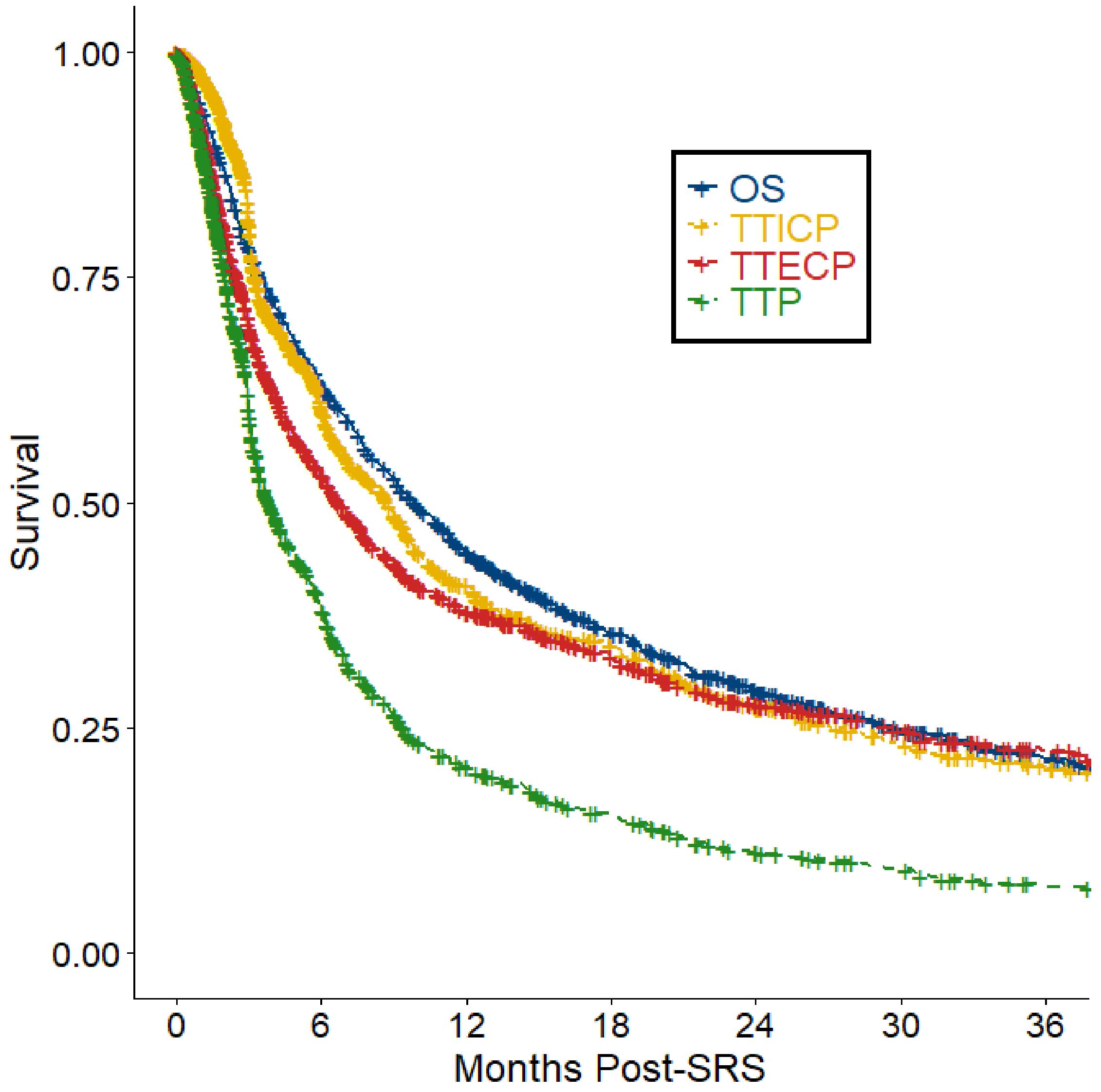

Number at risk

	0	6	12	18	24	30	36
OS	1383	830	553	377	271	189	132
TTICP	1383	547	279	186	118	82	57
TTECP	1383	536	308	211	141	96	65
TTP	1383	396	183	119	73	52	33

Months Post-SRS

© 2023 Carpenter DJ et al. *JAMA Network Open*.

eTable. Crude Event Rates for Death, Intracranial Progression (ICP), and Extracranial Progression (ECP) Across All Patients

		Death	
		Yes, No. (%)	No, No. (%)
**ICP**	Yes	492 (36%)	206 (15%)
	No	508 (37%)	177 (13%)
**ECP**	Yes	627 (45%)	173 (13%)
	No	373 (27%)	210 (15%)
		**ICP**	
		Yes, No. (%)	No, No. (%)
**ECP**	Yes	482 (35%)	318 (23%)
	No	216 (16%)	367 (27%)

© 2023 Carpenter DJ et al. *JAMA Network Open*.
