# Supplementary material for: Intracranial and Extracranial Progression and Their Correlation With Overall Survival After Stereotactic Radiosurgery in a Multi-institutional Cohort With Brain Metastases
Source: JAMA Netw Open. 2023 Apr 26;6(4):e2310117. doi: 10.1001/jamanetworkopen.2023.10117 (PMC10134007; doi:10.1001/jamanetworkopen.2023.10117)
Supplement: Supplement 2. — Data Sharing Statement [file jamanetwopen-e2310117-s002.pdf]

Data Sharing Statement

Carpenter. Intracranial and Extracranial Progression and Their Correlation With Overall Survival After Stereotactic Radiosurgery in a Multi-institutional Cohort With Brain Metastases. *JAMA Netw Open*. Published April 26, 2023. doi:10.1001/jamanetworkopen.2023.10117

Data
Data available: No
